# Definition of the *To Be Named Ligament* and *Vertebrodural Ligament* and Their Possible Effects on the Circulation of CSF

**DOI:** 10.1371/journal.pone.0103451

**Published:** 2014-08-01

**Authors:** Nan Zheng, Xiao-Ying Yuan, Yun-Fei Li, Yan-Yan Chi, Hai-Bin Gao, Xin Zhao, Sheng-Bo Yu, Hong-Jin Sui, John Sharkey

**Affiliations:** 1 Department of Anatomy, College of Basic Medicine, Dalian Medical University, Dalian, P. R. China; 2 Department of Anatomy, Zhongshan College of Dalian Medical University, Dalian, P. R. China; 3 Dalian Hoffen Bio-Technique Co. Ltd., Dalian, P. R. China; 4 School of Art, Dalian Medical University, Dalian, P. R. China; 5 National Training Centre, University of Chester, Dublin, Ireland; University of Palermo, Italy

## Abstract

Few studies have been conducted specifically on the dense connective tissue located in the posterior medial part of the cervical epidural space. This study was undertaken to examine the presence of this connection between the cervical dura mater and the posterior wall of spinal canal at the level of C1–C2. 30 head-neck specimens of Chinese adults were used. Gross dissection was performed on the suboccipital regions of the 20 specimens. Having been treated with the P45 plastination method, 10 specimens were sliced (9 sagittal and 1 horizontal sections). As a result, a dense fibrous band was identified in the nuchal ligament of 29 specimens (except for one horizontal section case). This fascial structure arose from the tissue of the posterior border of the nuchal ligament and then projected anteriorly and superiorly to enter the atlantoaxial interspace. It was termed as ***to be named ligament*** (TBNL). In all 30 specimens the existence of a fibrous connection was found between the posterior aspect of the cervical dura mater and the posterior wall of the spinal canal at the level of the atlas to the axis. This fibrous connection was identified as ***vertebrodural ligament*** (VDL). The VDL was mainly subdivided into three parts, and five variations of VDL were identified. These two structures, TBNL and VDL, firmly link the posterior aspect of cervical dura mater to the rear of the atlas-axis and the nuchal region. According to these findings, the authors speculated that the movements of the head and neck are likely to affect the shape of the cervical dural sleeve via the TBNL and VDL. It is hypothesized that the muscles directly associated with the cervical dural sleeve, in the suboccipital region, may work as a pump providing an important force required to move the CSF in the spinal canal.

## Introduction

Over the past 30 years, the suboccipital region has received an increasing amount of attention in the scientific literature. Research has revealed multiple soft-tissue connections extending from the suboccipital structures to the cervical dura mater through the posterior intervertebral spaces [1]–[5]. Thus, these connections can be regarded as fibrous fascial tissue bridges providing a continuity of the suboccipital muscles, integrating motion of the atlantooccipital and cervical intervertebral joints with that of the cervical dura mater [2], [5]–[8]. All of these reports clearly show that the suboccipital region is one of the most complex anatomical regions in the human body. In this region, the associated fascia of the rectus capitis posterior minor (RCPmi) continues anteriorly to contribute to the atlantooccipital myodural bridge. This then fuses with the posterior atlantooccipital membrane continuing on to merge with the cervical dura mater [2], [3]–[5], [9], [10].

The RCPmi, the rectus capitis posterior major (RCPma) and the obliquus capitis inferior (OCI) all contribute to the atlantoaxial myodural bridge. This bridge travels through the posterior atlantoaxial interspace and links these muscles to the cervical dura mater [1], [5], [8], [11]–[15].

Aside from suboccipital muscles, the nuchal ligament (NL) has been shown to attach to the cervical dura mater via the atlantooccipital and atlantoaxial interspaces [3], [16], [17]. However, these findings have been disputed. A study reported [18] that ventral portions of the NL was loose above the level of C2 due to the presence of fatty tissue and therefore it was difficult to discern a midline NL structure above that level.

In summary, in the posterior epidural space of the cervical vertebral canal there is a dense fascial tissue band linking the posterior aspect of the cervical dura mater to the posterior apparatus of the vertebral canal. This fascial tissue continues on to attach to the suboccipital structures via the atlantooccipital and atlantoaxial interspaces. This connective tissue system integrates motion of the craniovertebral and cervical intervertebral joints with that of the cervical dura mater. In 1929 Von Lanz [19] described the ligamentum craniale durae matris spinalis (CDMS ligament) as a series of fibrous strands lying between the dura mater and the apparatus of the vertebral canal. We could find no study conducted specifically on the linking apparatus in the posterior midline of the epidural space integrating the motion of the head and neck with the cervical dura mater. The objective of this study is to examine the presence of this fascial connection in the posterior epidural space at the level C1 to C2 and the contribution of the NL to this fascial connection. Additionally, we aim to examine this fascial connection’s course and composition from the perspective of its gross and sectional anatomy.

## Materials and Methods

### Ethics statement

This study was approved by the ethics committee of the Body and Organs Donation Center of Dalian Medical University. The research involved thirty head-neck specimens of Chinese adults in middle and old age. The specimens were from the Body and Organs Donation Center of Dalian Medical University. Written informed consent was obtained from the donors involved in this study prior to death in accordance with the regulation of the ethics committee.

The specimens were preserved using a formalin-alcohol mixture. Twenty of the specimens (five female and fifteen male) were dissected. Ten male specimens were sliced by P45 sheet plastination technique for the purpose of this study (nine in sigttal section and one in horizontal section). Specimens with evidence of cervical surgery or trauma were excluded from this study. Photographic documentation was recorded with a Canon EOS 450D camera, using a Canon EF-S 18–55 mm f/3.5–5.6 IS zoom lens.

### Dissection of the suboccipital region

Each dissection began with the removal of soft-tissue structures superficial to the vertebral column, ensuring the RCPmi and the NL remained intact ensuring preservation of the area of interest. Careful observation was focused on the structures of the atlantoaxial interspace and its relationships with the NL and the RCPmi. Using a Stryker Autopsy 810 saw (Stryker, Kalamazoo, MI), gross anatomical cuts were then performed bilaterally along the posterior arch of the atlas (C1) and the lamina of the axis (C2). Using a surgical scalpel, the RCPmi was detached from the inferior nuchal line. Following these procedures, the posterior arch of the atlas and the lamina of the axis were reflected backwards or towards the lateral side to reveal the contents of the vertebral canal, especially the structures situated between the dura mater and the posterior wall of the vertebral canal.

### P45 sheet plastination of the head and neck

In ten specimens of the head-neck, nine were sliced in sagittal section, while one was sliced in horizontal section. The P45 sheet plastination is a relatively new patented technology in china [20]. P45 sheet plastination is a special polyester resin corrosion method designed to preserve biological sectional specimens in situ. The P45 plastination sheet provides good light transmission, allowing the internal structure of the sheet to be revealed clearly and intact. The procedure of the P45 sheet plastination was as follows:

#### Slicing

The embalmed specimens of the head-neck were frozen at −70°C for two weeks and then embedded in polyurethane foam and frozen at −70°C, again for two days. After freezing, sagittal slices were made at 3 mm from side to side on a high-speed band saw. The volume of sawdust was 1 mm. The slices were placed in an orderly fashion on polyethylene grids with a piece of fly screen. A light stream of running water removed any sawdust. The grids were then stacked together and the twines tied to hold the grids as one unit. All the units were then labeled and put into square polyethylene pails.

#### Bleaching

All the slices were rinsed overnight in cold running water, following which the slices were immersed in 5% dioxogen overnight.

#### Dehydration

After bleaching, the slices were dehydrated by the freeze substitution method. First, the slices were precooled at 5°C in order to avoid the formation of ice crystals and shrinkage before being placed into cold acetone. The slices were then placed in the first bath of 100% acetone at −25°C for one week and then transferred into a second bath of 100% acetone at −15°C for ten days. Following this ten-day period they were then put into 100% acetone at room temperature for one week. The slices were finally submerged in fresh 100% acetone at room temperature. After one week, slices were taken out for impregnation. The purity of the acetone was monitored with an acetonometer on a daily basis. Provided the purity had remained similar on three monitorings, the slices were moved to a fresh dehydrating solution.

#### Casting and forced impregnation

After dehydration, the casting mold was prepared for the casting. It was a flat chamber consisting of two plates of 5 mm tempered glass, flexible 4 mm latex tubing, and several large fold back clamps. The slices were lifted out of the acetone bath and placed between two glass plates. The molds were then filled with polyester (Hoffen polyester P45, China) via a funnel. The components of Hoffen polyester P45 were mixed at 1000 ml of polyester P45 monomer to 10 g of P45a to 30 ml of P45b to 5 g of P45c. The P45a and P45c were used as plasticizer, and the P45b was a hardener for sheet plastination.

On completion of casting, the filled mold was placed upright into a vacuum chamber, at room temperature, for impregnation. Some larger bubbles were removed manually with a piece of 1 mm stainless steel wire. The absolute pressure was slowly decreased to 20 mm Hg, 10 mm Hg, 5 mm Hg, and 0 mm Hg according to the release of bubbles. The pressure of 0 mm Hg was maintained until bubbling ceased. Duration of impregnation was in excess of eight hours.

#### Curing

After the vacuum had been released, the air bubbles within the sheets were checked and removed. The alignment of the slices was checked and corrected using the steel wire. The top of the mold was clamped with large fold back clamps, and the sheet was then ready for curing.

The sheets were cured using heated bath water. The sheets were placed upright in the bath water at 40°C for 3 days. In order to equilibrate the temperature of the water a small circulatory pump was used to circulate the water around the bath.

#### Cutting and sanding the molds

After curing, the sheets were removed from the bath and cooled to room temperature in a rack. The slices were then taken from the flat chamber and were covered appropriately with adhesive plastic wrap for protection. A bend saw was used to cut and trim the plastic along the edges about 1 mm outside of the slices. Following this, a wool sander was used to remove the sharp edges of the slices and after sanding, the adhesive plastic wraps were removed and the slices were put into plastic wraps to avoid scratches. The head-neck sheets were then observed and photographed on the fluorescent film-reading lights.

## Results

### *To Be Named Ligament* (TBNL)

**Figure 1:**
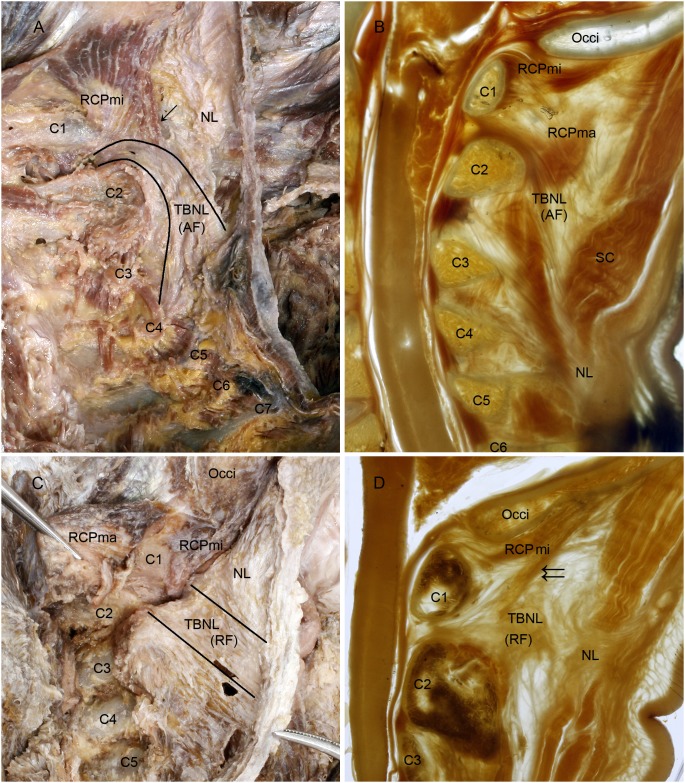
The *to be named ligaments* were shown in the dissected specimens and the P45 plastination sheets. A, C: Lateroposterior aspect of the nuchal ligament; B, D: Median sagittal section of the head-neck in a P45 plastination sheet. *To Be Named Ligament* (TBNL) within the nuchal ligament was formed by arcuate fibers (A, B). The arcuate fibers arose from the lower part of the posterior border of NL below the level of the spinal process of vertebra C3 (A, B), continuing anterosuperiorly crossing over the spinous process of axis and continuing into the atlantoaxial interspace (B). Its path continues traversing through the interspace and attaching to the posterior aspect of the cervical dura mater (B). The rectus capitis posterior minor (RCPmi) emitted a bundle of muscular fibers (↓) which were attached to TBNL (A). Another kind of TBNL within the nuchal ligament was formed by radiated fibers (C, D). The radiated fibers originated from the posterior border of the upper part of nuchal ligament opposite to the spinal processes of vertebrae C2 and C3. At this point the radiated fibers ran anteriorly to traverse through the atlantoaxial interspace and finally attached to the posterior aspect of the cervical dura mater (D). Additionally, the RCPmi emitted a bundle of muscular fibers (↓↓), which merged into TBNL before it enterd the atlantoaxial interspace (D). TBNL, *To Be Named Ligament*. NL, nuchal ligament. AF, arcuate fibers of TBNL. RF, radial fibers of TBNL. C1∼C7, first to seventh cervical vertebra; Occi, occipital bone; RCPma, rectus capitis posterior major; SC, splenus capitis.

From the twenty head-neck specimens dissected, 20 nuchal ligaments (NLs) were completely isolated from the muscles of the nuchal region. From the ten head-neck slices, 9 NLs were shown in the P45 plastination sheets, except for the one specimen in horizontal section. In each of the 29 NLs, a dense fibrous band was clearly identified arising from the tissue of the posterior border of NL projecting anteriorly and superiorly to enter the atlantoaxial interspace (Figure 1. A, C). It then traversed through the interspace and attached to the posterior aspect of the cervical dura mater between the first and second cervical vertebrae (Figure 1. B, D). This fibrous band within NL had never been named before, so we referred to it as the ***to be named ligament*** (TBNL).

As a dense part of NL, the TBNL was formed by either arcuate or radiated fibers. In 22 of 29 specimens the arcuate fibers of TBNL arose from the lower part of the posterior border of NL below the level of the spinal process of the vertebra C3, where the common origins of the splenius capitis, superior posterior serratus and rhomboid minor muscles can be seen (Figure 1. A, B). The arcuate fibers then ran anterosuperiorly crossing over the spinal process of axis to continue into the atlantoaxial interspace (Figure 1. A, B). Unlike the arcuate fibers, the radiated fibers of TBNL in 7 of 29 specimens arose from the upper part of the posterior border of NL just opposite to the spinal process of the vertebrae C2 and ran anteriorly and straight within NL inserting into the atlantoaxial interspace (Figure 1. C, D).

Additionally, in some specimens the RCPmi emitted a bundle of muscular fibers that did not terminate at the posterior arch of the atlas but instead terminated at TBNL before the TBNL entered the atlantoaxial interspace. With further examination, it was shown that this bundle of muscular fibers had two ways in which to arrive in TBNL. One was away from the atlantoaxial interspace along the TBNL (Figure 1. A) and the other was entering the interspace along with the TBNL (Figure 1. D). It is assumed that, based upon the termination of RCPmi, RCPmi might affect the function of TBNL in some manner.

### *Vertebrodural Ligament* (VDL)

**Figure 2:**
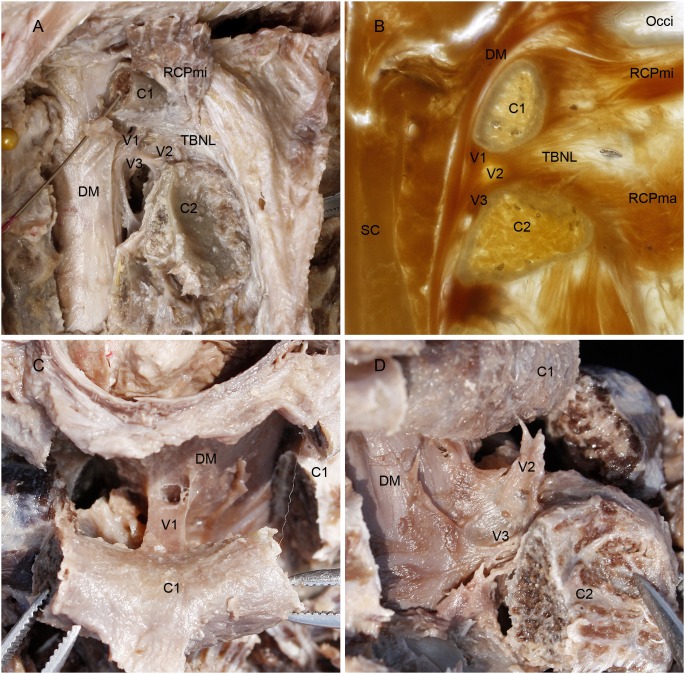
V*ertebrodural Ligaments* as shown in the dissected specimens and the P45 sheet plastination. A: Posterolateral aspect of the superior cervical vertebral canal; B: Median sagittal P45 plastination sheet of the superior cervical vertebral canal; C: Superior aspect of the dura mater and the posterior arch of atlas; D: Posterolateral aspect of the dura mater and the atlantoaxial interspace. On dissecting the posterior arch of the atlas and the lamina of the axis bilaterally and reflecting from one side to another (A), the *vertebrodural ligament* (VDL) was exposed in the posterior epidural space, which connected the dura matter with the atlas, axis and atlantoaxial space, thus the VDL was subdivided into three parts: the atlantal part, the TBNL’s part and the axial part (A). The median sagittal P45 plastination sheet of the superior cervical vertebral canal also showed that VDL consisted of these three parts (B). These three parts of VDL merged with each other and ran down almost vertically in the posterior epidural space continuing to the dura mater just anterior to the lamina of the axis (A, B). When the posterior arch of the atlas was cut bilaterally and reflected posteriorly, the atlantal part of VDL was shown specially in superior aspect, which was coronally banded in shape (C). When the lamina of the axis was cut bilaterally and then reflected posteroinferiorly, the TBNL’s and axial parts of VDL were shown in a lateral view to protrude from the dura mater in the median sagittal position and extending to the atlantoaxial interspace and the lamina of axis (D). VDL, the *vertebrodural ligament*. V1, atlantal part. V2, TBNL’s part. V3, axial part. RCPmi, rectus capitis posterior minor; C1, posterior arch of atlas; TBNL, *to be named ligament*; C2, lamina of vertebral arch of axis; DM, dura mater; Occi, occipital bone; SC, spinal cord.

In each of the thirty head-neck specimens observed, including twenty suboccipital dissections and the ten sets of P45 plastination sheets, the existence of a fibrous connection was found between the posterior aspect of the dura mater and the posterior wall of the spinal canal from the atlas to the axis (Figure 2. A, B). With further examinations, it was demonstrated that the complex of dense connective tissue completed these connections. According to its origin and termination, it was termed the ***vertebrodural ligament*** (VDL).

The VDL is a dense connective tissue complex extending from the posterior arch of the atlas, the atlantoaxial interspace and the lamina of the axis to the dura matter. Because its origin was broad from the atlas to the axis, the VDL is mainly subdivided into three parts as follows (Figure 2. A, B).

#### The superior portion was identified as the atlantal part (V1)

It originated from the median area of inferior border of the anterior aspect of the posterior arch of the atlas. It was banded in shape and located in a coronal position (Figure 2. C).

#### The middle portion was identified as the TBNL’s part (V2)

This portion originated from the atlantoaxial interspace. It was continuous with the TBNL and located in the median sagittal position (Figure 2. D).

#### The inferior portion we called the axial part (V3)

It originated from the midline of the superior part of anterior aspect of the lamina of the axis. It was the lower extending of the TBNL’s part, which adhered to the lamina and became the connection between the axis and the dura mater (Figure 2. D).

**Figure 3:**
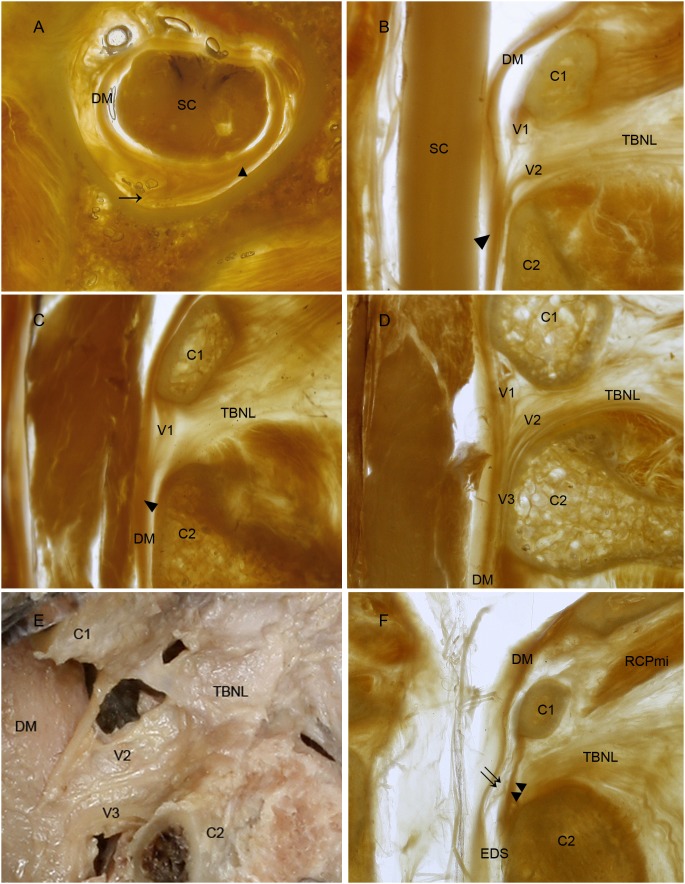
VDL has five types according to its different components. The *vertebrodural ligament* is located in the posterior epidural space at the level from atlas to axis (A). It is inserted into the dura mater providing the dura matter with additional thickness (A). On rotating the neck, the *vertebrodural ligament* draws the posterior part of the dura mater, slightly, to the lateral side. According to its various components, VDL was observed as comprising of five specific types. Type I was the balance type. This was the strongest type, which was thick in each of the three parts (Figure 2 A, B). Type II was the secondary balance type with the V3 weaker or absent (B). Type III was the atlas dominant type. In this type, V1 was thicker while V2 and V3 were weaker (C). Type IV was the TBNL dominant type, in which V2 was stronger than the others (D, E). Type V was the weakest type with few connective fibers extending from the posterior wall of the spinal canal to the cervical dura mater. In this kind of specimen, the atlantoaxial interspace was closed by the atlantoaxial membrane (or yellow ligament), and therefore the TBNL could not extend from the interspace into the epidural space as a result of weakness of the VDL (F). ↑, *vertebrodural ligament*. ▴, the thick area of dura matter. DM, dura mater. SC, spinal cord. C2, axis. V1, atlantal part. V2, TBNL’s part. V3, axial part. TBNL, *to be named ligament*. EDS, epidural space. ↑↑, weakness of the VDL. ▴▴, the atlantoaxial membrane (or yellow ligament).

Generally, the V1 and V2 parts were dense while the V3 was loose. Finally, all parts merged with each other and formed a strong VDL (Figure 2. A, B). The VDL ran down almost vertically in the posterior epidural space and continued to the dura mater just anterior to the lamina of the axis (Figure 2. B, Figure 3. A). At the region of the VDL insertion, the dura mater became distinctly thick (Figure 3. A, B, C). It was, therefore, indicated that the VDL was attached firmly to the posterior aspect of the dura mater.

From the 29 specimens (except for one case, it was not possible to clearly identify a whole VDL as a result of the horizontal section), five variations of VDL were found according to its different components. Type I was the balance type, found in 17 of 29 specimens. This was the strongest type, which was thick in each of the three parts (Figure 2. A, B). Type II was the secondary balance type with the V3 weaker or absent (Figure 3. B), found in 1 of 29 specimens. Type III was the atlas dominant type, found in 5 of 29 specimens. In this type, V1 was thicker while V2 and V3 were weaker (Figure 3. C). Type IV was the TBNL dominant type, in which V2 is stronger than the others (Figure 3. D, E), found in 4 of 29 specimens. Type V was the weakest type with few connective fibers extending from the posterior wall of the spinal canal to the cervical dura mater, found in 2 of 29 specimens. In this kind of specimen, the atlantoaxial interspace was closed by the atlantoaxial membrane (or yellow ligament), and therefore the TBNL could not extend from the interspace into the epidural space as a result of weakness of the VDL (Figure 3. F).

## Discussion

Our study reports the presence of the *to be named ligament* (TBNL) and *vertebrodural ligament* (VDL) in the suboccipital region and posterior epidural space. The TBNL is a local enhancement of the NL, the dense fibers, which extends from the posterior border of the NL into the atlantoaxial interspace. The VDL is situated in the posterior epidural space at the level from the atlas to the axis, as a dense connection, which attaches the cervical dural mater firmly to the posterior wall of the vertebral canal. The TBNL is an anatomical continuity of the VDL.

To our knowledge, the literature has not described either the VDL or the TBNL and their continuity until now. In our study, these two structures were present in all samples. Pathological signs, such as inflammation and adhesions, were not found and therefore we consider that they are normal, nonpathological structures in human beings. Our photographic data revealed that these two structures were present in both dissected specimens and P45 plastination sheets. A gross dissection is helpful to understand a structure as a whole and its local and possible distal relationships. A sheet plastination technique P45 is a special method for transparent sectional specimens, which has a great advantage of displaying fibers of connective tissue and myofascial structures in situ [20]. Using these methods, especially the latter, we can ensure the results are extremely accurate.

Since 1998, studies have shown that the NL attaches to the cervical dura mater. These studies noted that NL attached to the cervical dura mater via a ligamentous connection through the atlantooccipital and atlantooccipital interspaces [3], [16], [17]. In 2000, Johnson et al. [18] reported that ventral portion of the NL did not become apparent until below the level of C2, and that it consisted of layers of vertically orientated fibers. These findings show that the dense connective tissue fibers do not distribute evenly in the NL. However, none of these studies mentioned which part of NL extends into the atlantooccipital interspace. Firstly, this study’s finding confirmed the connection between the NL and the cervical dura mater. Secondly, only our results revealed the presence of a dense portion in the NL, termed the *to be named ligament* (TBNL), which was located in the anteroinferior or middle part of the NL within the median sagittal plane, coursed through the atlantooccipital interspace and ultimately attaching to the cervical dura mater. The course of fibrous strands of the TBNL was shown clearly, especially, in the plastination sheets technique. Showing the fiber’s course is crucially helpful in understanding how the NL exerts a force on the cervical dura mater. The TBNL originates from the posterior border of the NL below the level of axis, especially from the “decussation of tendons” of the trapezeus, splenius capitis, and rhomboideus minor. It then extends anterosuperiorly or anteriorly into the atlantoaxial interspace to take part in forming the connection between the cervical dura mater and the posterior wall of the vertebral canal. Therefore, we can speculate that TBNL is stretched tight when we nod. As a result, the TBNL can draw the cervical dura mater posteriorly via the atlantooccipital interspace. Additionally, it was shown in our study that RCPmi might emit a muscular bundle terminating at TBNL. It is assumed that RCPmi might provide essential functional forces on the cervical dura mater via the TBNL. A detailed description of RCPmi’ terminating at TBNL will be performed in another investigation. Furthermore, it is a logical inference that any structure connected firmly with the TBNL could exert a force resulting in a potential effect on the dura mater. We will fully explore this in a follow-up study.

Concerning connections between the spinal dura mater and the vertebral canal, the history of studies can be traced back more than 100 years. In the years 1888 to 1898, it had been depicted that the spinal dura mater was anchored to the inner surface of the vertebral canal wall by numerous and thin segmental fibers, namely meningovertebral ligaments, which occurred consistently in the cervical region [21], [22]. Furthermore, in the upper cervical region and the atlantooccipital joint, the range of motion between vertebrae is greater than that in any other region, so another extensive fibrous connective tissue would exist in the epidural space to provide anchoring of the dura mater to the vertebral canal. In 1929 the CDMS (**craniale durae matris spinalis**) ligament was described as a series of fibrous strands lying between the cervical dura mater and the edge of the foramen magnum, the posterior border of the atlantooccipital joints, the posterior arch of the atlas, and the arch of the axis [19]. A series of observations published recently supported and supplemented the descriptions of the CDMS ligament. Lang [23] mentioned the CDMS ligament but omitted reference to the connection between the dura and the arch of the axis. Rutten et al. [12] described a transverse strand of up to 9 mm in width attaching the dura to the ventrocaudal side of the posterior arch of the atlas. Rutten observed that the medial part of this band was continuous with the deep part of the NL. Moreover, Mitchell et al. [16], Dean et al. [17] and Humphreys et al. [3] described a direct continuity between the NL and the spinal dura at the junction between C1 and C2. The CDMS ligament has an abundance of contents that have been well described in anatomy [3], [12], [16], [17], [19], [23], however, there are still some questions to be solved. One of these questions concerns the relationship that exists among the series of fibrous strands in CDMS. In this study, three fibrous bands were found in the posterior-median part of the epidural space in the upper cervical region. The first band originates from the ventrocaudal side of the posterior arch of the atlas, the second one originates from the terminal part of the TBNL in the atlantoaxial interspace and the third one originates from the lamina of the axis respectively. These findings are similar to those described previously [3], [16], [17], [22] confirming the linking between the dura mater and the posterior apparatus of the vertebral canal. However, only our study, reports that these three bands fused with each other as a powerful single unit inserted to the posterior aspect of the cervical dura mater. It was named as *VertebroDural Ligament* (VDL) in the present study. The VDL firmly linked the dura mater to the posterior wall of the spinal canal from the atlas to the axis. Additionally the VDL consistently orients ventro-vertically and terminates at the posterior aspect of the dura mater just anterior to the lamina of axis. Furthermore, the proportion of each part of VDL varies significantly among the specimens. This being the case, five types of the VDL were presented in our study accordingly.

It is highly possible that in the upper cervical region, the VDL serves as a vehicle to integrate the motion of the head and neck with the cervical dura sleeve. When a person nods his or her head, the TBNL can draw the posterior aspect of the sleeve posteriorly via the VDL; and when a person rotates his or her head, the atlas can draw the posterior aspect of the sleeve laterally via the VDL.

Moreover, recent studies have depicted fibrous conjunctive tracts that extend from the anterior fascia of the RCPma and OCI muscles, spanning through the atlantoaxial interspace, and ultimately connecting to the cervical dura in the vertebral canal [1], [5], [14]. Additionally, plastinated sectional observation by Yuan et al. (to be submited in another paper in 2014) revealed that the RCPmi partially terminated at the atlantoaxial interspace. On integrating the findings of these literatures with our results, it seems reasonable to suggest that the Suboccipital Myodural Bridge through the atlantoaxial interspace is part of the VDL and that the related suboccipital muscles may directly exert a force on the cervical dura mater via the VDL. Moreover, Scali et al. [8] reported the presence of a posterior dural bulge at C1–C2 in a significant sample size of the MR image population, for which the physiological contraction of the suboccipital muscles and the movements of the head and neck may be a reasonable explanation. These possible correlations require further investigation.

## Conclusions

Our study reports the presence of two new normal structures from the perspective of the gross and sectional anatomy. They are located in the NL and the posterior cervical epidural space, namely TBNL and VDL respectively. These two structures firmly link the posterior aspect of cervical dura mater to the rear of atlas-axis and the nuchal region. According to this finding, the authors speculate that the movements of the head and neck are likely to affect the shape of the cervical dural sleeve via the TBNL and VDL in some mechanical or kinematic manner, and as a result, will affect the volume of the subarachnoid space in the upper cervical part. Further speculation also seems to be reasonable. The movements of the head and neck are likely to contribute to the circulation of cerebral spinal fluid. Moreover, the muscles-VDL-cervical dural sleeve complex in the suboccipital region might work as a pump to provide an important part of the circulating power to the CSF in the spinal canal. This bold hypothesis will be further investigated in our follow-up studies.
